# The Effects of Nanohydroxyapatite with and without Low-Power Laser and Cold Atmospheric Plasma on Enamel Remineralization: *in vitro* Study 

**DOI:** 10.30476/dentjods.2024.102452.2363

**Published:** 2025-09-01

**Authors:** Pegah Bakhtiary, Matine Gharavi, Hooman Ebrahimi, Katayoun Salem

**Affiliations:** 1 Dept. of Pediatric Dentistry, Faculty of Dentistry, Tehran Medical Sciences, Islamic Azad University, Tehran, Iran.; 2 Postgraduate Student, Dept. of Pediatric Dentistry, Faculty of Dentistry, Tehran Medical Sciences, Islamic Azad University, Tehran, Iran.; 3 Oral Medicine Specialist, Fellowship in Orofacial Pain, Fellowship in Laser in Dentistry, Researcher, Oral Medicine Department, Faculty of Dentistry, Tehran Medical Sciences, Islamic Azad University, Tehran, Iran.; 4 Dept. of Pediatric Dentistry, Faculty of Dentistry, Tehran Medical Sciences, Islamic Azad University, Tehran, Iran. Second Affiliation University of Applied Science and Technology, EbneSina Center, Tehran, Iran.

**Keywords:** Cold Plasma, Diode Laser, Fluorides, Hydroxyapatites, Hardness Test

## Abstract

**Background:**

Remineralizing early enamel lesions in primary dentition remain a significant challenge in dentistry, requiring innovative approaches to enhance enamel repair.

**Purpose:**

This study aimed to evaluate the efficacy of a commercially available nanohydroxyapatite paste (Nano P), alone and in combination with air dielectric barrier discharge cold atmospheric plasma (CAP) and a 940-nm diode laser, on enamel remineralization.

**Materials and Method:**

In this *in vitro* study, 105 intact anterior primary teeth were randomly assigned into one control and six experimental groups: (I) control, (II) Fluoride varnish, (III) Nano P, (IV) Nano P + dye + laser, (V) Nano P + laser, (VI) CAP, and (VII) Nano P + CAP. Caries-like lesions were induced by demineralization. Microhardness was evaluated before and after demineralization and 4 weeks post-remineralization with pH cycling. Data analysis was conducted using one-way ANOVA (α=.05) and Tamhane’s Post-Hoc test, with effect sizes determined by Cohen’s d test and Hedges' g correction. The percentage of recovery of the enamel microhardness was calculated

**Results:**

Initial and secondary microhardness were similar across groups (*p* Value >.05). Post-remineralization, significant differences were observed (*p*< 0.05), with the highest microhardness in the Nano P+ laser group, followed by Nano P+ dye+ laser, Nano P+ CAP, Nano P, fluoride varnish, CAP, and the control. Pairwise comparisons showed significant differences between all pairs except Nano P+ laser and Nano P+ dye + laser (*p*= 0.7, Effect size=0.4).

**Conclusion:**

Nano P combined with a laser, followed by Nano P with plasma, and Nano P alone significantly increased microhardness more than fluoride or plasma, suggesting these combinations as effective alternatives for enamel remineralization in primary teeth.

## Introduction

Dental decay progresses through a continuum of demineralization and remineralization of enamel, initially reversible but advancing through stages from normal clinical appearance to non-cavitated enamel decay, microscopic cavitated lesions, and ultimately clear dentin decay. Various methods exist to intervene in this process to either halt demineralization or initiate remineralization [ 1 - 3 ].

In recent decades, efforts to enhance remineralization have primarily centered on fluoride compounds, recognized as the gold standard for decay control. However, their limitations in effectively remineralizing subsurface enamel have prompted exploration into alternative approaches, including non-fluoride compounds and their combinations with other substances or techniques [ 3 - 7 ]. Fluoride functions by preventing mineral loss and promoting remineralization [ 3 - 7 ].

Emerging substances such as nano-hydroxyapatite have attracted attention for their ability to mimic biological hydroxyapatite and effectively rebuild demineralized enamel, potentially surpassing fluoride in their efficacy as anti-decay agents . Laser therapy presents another promising avenue by inducing chemical modifications in enamel that enhance resistance to decay and facilitate increased fluoride absorption and remineralization [ 10 - 17 ]. Notably, low-power diode lasers, valued for their affordability and portability, have shown promise in preventing enamel demineralization, although their effectiveness in remineralizing initial enamel lesions requires further investigation [ 17 ].

More recently, cold atmospheric plasma has emerged as an innovative method for controlling dental decay. Plasma, an intermediate gaseous substance, demonstrates the capability to penetrate irregularities and cavities, effectively eliminating pathogens within dental plaque and decay without causing thermal discomfort or adverse effects [ 18 - 22 ]. Its versatility in various dental applications, including implant repair, adhesion, endodontic treatments, and tooth whitening, stems from its physical, chemical, and biological interactions with compounds, preserving structural integrity [ 19 - 22 ].

Considering the existing gaps in research concerning adjunctive methods with remineralizing compounds, this *in vitro* study aims to evaluate the efficacy of a nano -hydroxyapatite-containing substance both independently and in conjunction with a 940-nanometer wavelength diode laser and cold atmospheric plasma for remineralizing initial enamel lesions in primary teeth. This research seeks to contribute insights into optimizing dental treatments by leveraging innovative approaches to enhance enamel remineralization beyond conventional fluoride-based methods. 

## Materials and Method

Considering α= 0.05, β= 0.20, a standard deviation of the mean microhardness equal to 30, and an effect size of 0.43, the minimum sample size required for each study group is determined to be 12 samples. For a more accurate evaluation, the number of samples in each group was set to 15. Hence, a total of 105 intact anterior teeth in one control and six experimental groups were evaluated [ 10 ].

Extracted teeth were disinfected in 0.5% chloramine T and stored in distilled water at 37°C until the start of the experiments. Anterior maxillary and mandibular teeth were evaluated for any defects, cracks, decay, wear, or discoloration under a stereomicroscope (ZSM 1001 model, Zist Rahe Danesh Company, Iran) at 40× magnification and included in the study.

The teeth used in this study were already extracted for reasons unrelated to this research, such as orthodontic treatment or other dental procedures, and were subsequently collected. This approach ensures the ethical use of human tissues and avoids unnecessary extraction of teeth solely for experimental purposes. Intact anterior teeth provide a consistent and uniform surface for evaluating enamel microhardness and the effects of different treatments, without the variability introduced by restorations or significant structural defects.

To conduct the experiments, first, the roots of the teeth were cut 2 millimeters below the enamel and cementum junction using a high-speed handpiece with air and water coolant (COXO, Foshan, China CX207-F H65). The teeth were then mounted in pink base plate wax (Base plate Wax, Keystone, Singen, Germany). To create a smooth and uniform enamel surface, the tooth surfaces were ground using silicon carbide sandpaper (Matador Starcke, Germany) with grit sizes of 600, 800, and 1200, followed by a slurry of alumina. Subsequently, the samples were rinsed with distilled water for 30 seconds and dried.

To determine the initial enamel microhardness, a 2×2-millimeter adhesive was placed on the buccal surface of the tooth, and the remaining areas of the buccal surface were covered with two layers of nail polish (Revlon, Brazil). Then, the adhesive was removed, and any excess materials were rinsed off with distilled water. A force of 50 grams was applied for 10 seconds using Vicker's diamond indenter (Wolpert, Darmstadt, Germany) on the isolated area. The Vicker's hardness number, expressed as the force in kilograms per square millimeter of the area, was calculated, and the average microhardness obtained at three points was reported as enamel microhardness [ 10 ].

The samples were allocated to 7 groups by simple computer-based random numbers. To create initial enamel lesions, teeth were immersed in a 30 mL demineralizing solution. The solution consisted of 50 mM acetic acid, 2.2 mM CaCl_2_·H_2_O, 2.2 mM KH_2_PO_4_, 1 M KOH, and traces of thymol (pH=4.4; 37°C), for 4 days. The pH was monitored daily and, if necessary, adjusted with small amounts of either 10% HCl or 10 M KOH to maintain a constant pH value [ 19 ]. After creating enamel lesions, the samples were rinsed with deionized water and assigned to study group.

### Group I (Control)

As the control group, no intervention was conducted for this group. The samples were immersed in artificial saliva for 24 hours and then subjected to the pH cycling process, which simulates oral conditions. The pH cycling process involved placing the samples in a demineralizing solution for 6 hours each day, followed by immersion in a remineralizing solution for 18 hours, with daily solution changes. This cycle was repeated for 5 days per week over 4 weeks [ 22 ]. 

### Group II 

Fluoride varnish 5% (FluoroDose®, Centrix Inc., Shelton, USA) was applied to the tooth surface using an applicator, as per the manufacturer's instructions. It remained on the sample surface for 6 hours and was then gently removed from the surface using cotton-tip applicators dipped in deionized water, without scrubbing. In contrast to other materials, the fluoride varnish has a sticky texture; the samples were examined under a stereomicroscope (ZSM1001 model, Zist Rahe Danesh Company, Iran) to ensure the removal of any remnants of varnish on the tooth surface. Laboratory technicians examined the samples under a stereomicroscope (ZSM1001 model, Zist Rahe Danesh Company, Iran). The samples were exposed to artificial saliva for 24 hours before pH cycling. The fluoride varnish group was considered as the standard group for comparison. 

### Group III 

Nano-hydroxyapatite paste (Nano-P) (Desensibilize Nano P, FGM, Joinville, SC, Brazil) was dispensed in a Dappen pot and scrubbed for ten seconds using a felt disk (FGM, Brazil) with a low-speed handpiece from the FGM Company (Joinville, SC, Brazil). It was allowed to rest on the tooth for 5 minutes. Then the excess was removed with a moist cotton pellet. The samples were rinsed with deionized water, exposed to artificial saliva for 24 hours, and then subjected to the pH cycling process, following the same protocol as Group I [ 23 - 24 ]. 

### Group IV

First, similar to Group III, Nano-P was applied on the tooth surface. Then, a diode laser (EzLase, Biolase, San Clemente, CA, USA) (wavelength 940nm, power 0.4 W, contact spot area 0.8cm², time 10s, Ed 2.5 J/cm²) was applied perpendicularly to the tooth surface at a distance of 1 millimeter, fixed on the Nano-P area. The samples were exposed to artificial saliva for 24 hours and then subjected to the pH cycling process, following the same protocol as Group I [ 25 ]. 

### Group V

First, Nano-P under conditions similar to Group III was combined with a blue edible dye (Wiltom, Iran). This was done to increase the absorption of the diode laser and resorb heat to avoid pulp and dentin damage. Then, the diode laser was applied under conditions similar to Group IV. The samples were exposed to artificial saliva for 24 hours and then subjected to the pH cycling process, following the same protocol as Group I [ 25 - 26 ]. 

### Group VI

Dielectric barrier discharge cold atmospheric plasma (CAP) (Dentopanel, Fanavaran Sepidjamgan Company, Tehran, Iran) was applied with a voltage of 220 volts, a maximum current of 3.0 amperes, a maximum power of 66 volts-amps, a time of 20 seconds, and a frequency of 10 kilohertz. The samples were exposed to artificial saliva for 24 hours and then subjected to the pH cycling process, following the same protocol as Group I [ 27 - 36 ]. 

### Group VII

Nano-P, similar to Group III, was used. Then, cold plasma was applied under conditions similar to Group VI. The samples were exposed to artificial saliva for 24 hours and then subjected to the pH cycling process, following the same protocol as Group I [ 37 - 38 ]. To simulate oral conditions, the samples were subjected to pH cycling for 28 days in the laboratory environment. In the present study, we did not use thermocycling for the aging purpose. The underlying reason was that material characteristics such as retention, or microleakage were not evaluated. However, thermocycling could be helpful but not necessary in similar studies [ 39 ].

The characteristics of the remineralizing agents and tools used in the study are presented in Table 1. 

**Table 1:** Characteristics of remineralizing agents/tools

Remineralizing Agent/ Tool	Brand	Composition
Sodium Fluoride	% FluoroDose®, Centrix Inc., Shelton, USA	5% Sodium fluoride varnish with xylitol, 22,600 ppm fluoride
Nano-Hydroxyapatite	Nano P, FGM, Joinville, SC, Brazil	Nano-particles of calcium phosphate incorporated into hydroxyapatite crystals, containing approximately 2% fluoride (9000 ppm), 10,000 ppm nano-hydroxyapatite, potassium nitrate, water, humectant, and flavor
Diode Laser	Biolase, USA	Wavelength 940 nm, power 0.4 W, contact spot area 0.8 cm2, time 10 s, Ed 2.5 J/cm2
Cold Plasma	Air dielectric barrier discharge cold atmospheric plasma (CAP), Fanavaran Sepidjamgan Company, Tehran, Iran	Voltage 220 volts, maximum current 3.0 amperes, maximum power 66 volts-amps, time 20 seconds, frequency 10 kilohertz

### The pH cycling

To simulate oral conditions, the samples were subjected to pH cycling for 28 days in the laboratory environment.

Samples were placed for 6 hours each day in a demineralizing solution containing 2mM calcium chloride, 2.2mM sodium dihydrogen phosphate, 0.05M acetic acid, 1M potassium hydroxide with a pH of approximately 5.4. Subsequently, for the remaining 18 hours of the day, the samples were placed in a remineralizing solution containing: 1.5mM calcium chloride, 9.0mM sodium dihydrogen phosphate, 0.15mM potassium chloride with a pH of 7. After 5 days of pH cycling, the samples were immersed in the remineralizing solution for 2 days (weekends), concluding the cycle. The solution was replaced daily [ 22 ].

By the completion of the pH cycling process, the samples were subjected to a microhardness test again using the same procedure described earlier to measure the post-treatment enamel microhardness.

To compare inter-group changes, the one-way AN-OVA test was utilized. Initially, Mauchly's test of sphericity was conducted. The purpose of this test was to determine the equality of variances, and due to the lack of sphericity, the Greenhouse-Geisser correction was employed. Since the data did not have homogeneity of variance, for the binary evaluations, the Tamhane’s post-hoc test was used. The percentage of recovery of the enamel microhardness (%REMH) was calculated to help understanding the exact effect of intervention considering both the baseline and the demineralization step in each group. Effect size was determined using Cohen’s to compare the magnitude of effect of significant findings.

### Ethical Considerations

In this study, ethical considerations were carefully addressed. Prior to commencing the research, the study title was registered with the university, and the thesis obtained ethical approval under code IR.IR.IAU. DENTAL.REC.1400.014. Integrity and honesty in the utilization of scientific resources were maintained throughout the study. 

## Results

Mauchly's test of sphericity was conducted and revealed a significant violation of variance equality (*p*< 0.0001). Therefore, the Greenhouse-Geisser correction (ε = 0.52) was applied for the ANOVA test. Overall, after remineralization, a significant difference was found between all groups (*p*< 0.001, F= 14.9).

As demonstrated in Table 2, during the first and second stages, there were no significant differences between groups in microhardness (MH). However, after remineralization, MH significantly increased in all groups (*p*< 0.000, F= 7.07), except the control group (which underwent only pH cycling). The descending order of increase from highest to lowest was (1) Nano-P + L, (2) Nano-P + L + dye, (3) Nano-P + P, (4) Nano-P, (5) Fluoride, (6) Plasma, and (7) Control. Using the Post Hoc test, the percentage of recovery of the enamel microhardness (%REMH) was calculated in each for group, which indicates a significant difference in %REMH between all groups (*p*< 0.000), except the two laser groups combined with Nano-P (*p*= 0.99).

**Table 2:** Comparison of sound, demineralized, and remineralized enamel microhardness between study groups

Group	Stage			
	Sound enamel	Demineralization	Remineralization	%REMH
	Mean ±SD	Mean ±SD	Mean ±SD	Mean ±SD
Control	371.71±71.18 ^a, A^	92.88 ±5.32^b, A^	78.22 ±4.32^c, A^	-5.18±2.12 ^d, A^
Fluoride	381.77 ±79.46 ^a, A^	91.43 ±4.70 ^b, A^,	134.35 ±16.12 ^c, B^	16.06±7.52 ^d, B^
Nano P	377.82±89.05 ^a, A^	93.32 ±4.95 ^b, A^	159.91 ±8.10 ^c, C^	25.48±7.97 ^d, C^
Nano P+ Laser	375.57 ±59.45 ^a, A^	93.10 ±4.62 ^b, A^	195.31 ±11.48 ^c, D^	37.17±11.18 ^d, D^
Nano P+ dye+ Laser	376.37 ±72.44 ^a, A^	93.30 ±5.14 ^b, A^	191.11 ±10.18 ^c, D^	36.96±11.25 ^d, D^
CAP	372.17 ±56.98 ^a, A^	92.94 ±S4.85D ^b, A^	95.76 ±18.27 ^c, E^	0.88±6.56 ^d, E^
Nano P+ CAP	371.40 ±53.07 ^a, A^	91.87 ±3.22 ^b, A^	174.93 ±13.73 ^c, F^	31.20±9.70 ^d, F^
*p* Value	0.85	0.35	<0.000	<0.000

Air dielectric barrier discharge cold atmospheric plasma (CAP). Statistical significance: *p* < 0.05. One-way ANOVA was used to compare microhardness measurements, measured in kg/mm^2^.

^a,b,c,d^: In each row, means with the same lowercase letter are not significantly different (within-group analysis).

^A,B,C,D^: In each column, means with the same capital letter are not significantly different (between-group analysis).

Statistical significance: *p* < 0.05

The effect size of the study groups in comparison to the control group was determined using Cohen’s d test. The results showed Nano-P combined with laser (with out and with dye) had the highest effect size (14.61 and 13.52 respectively). The effect sizes of other groups in descending order are Nano- P (12.71), plasma+Nano-P (9.57), fluoride (4.86), and plasma (1.34). 

## Discussion

The present *in vitro* study investigated the efficacy of a commercially available Nano-P alone and in combination with CAP and a low-power diode laser (940 nm) for remineralizing artificial enamel caries in primary teeth. Control, plasma-only, and fluoride varnish groups were included for comprehensive evaluation.

Our findings highlight those combinations involving Nano-P and laser therapy demonstrated superior remineralization effects compared to other treatments. Specifically, the Nano-P+ laser group exhibited the highest final microhardness, followed by Nano-P+ dye+ laser, Nano-P+ plasma, and Nano-P alone, in descending order. Both fluoride varnish and plasma-alone treatments also showed enhanced remineralization compared to the control group. Statistical analysis confirmed significant differences among all experimental groups, except between the Nano-P+ laser and Nano-P+ dye+ laser groups, which showed comparable outcomes.

The synergistic application of Nano-P and low-power diode laser therapy yielded a substantial effect size, consistent with previous studies by Ahrari *et al*. [ 17 ], and El Assal *et al*. [ 24 ]. These studies emphasized the efficacy of combining Nano-P with both low and high-power laser therapies in promoting dental remineralization in permanent teeth, with our study extending this understanding to primary teeth, which possess distinct structural and compositional characteristics.

The enhanced effectiveness of low-power diode lasers in conjunction with Nano-P can be attributed to their ability to maintain optimal temperatures (250 to 400°C) for enhancing enamel crystalline structure, facilitating favorable transformations without adverse effects such as cracking or protein denaturation [ 40 - 41 ]. Plasma treatment alone exhibited reduced effectiveness in our study, likely due to its abrasive properties and the potential erosion of enamel surface features caused by interactions with heavy particles [ 31 - 33 ].

A noteworthy advantage of low-power lasers in the context of enamel remineralization, as opposed to high-power lasers, is their capacity to preserve the enamel's organic matrix. Low-power lasers, by sealing off diffusion channels in the enamel matrix between the rods, prevent ion exchange. This approach not only augments the enamel's resistance to demineralization but also contributes to the longevity of the remineralization effects [ 42 - 43 ].

Furthermore, the composition of primary enamel, characterized by higher organic content and distinct mineral distribution, influences its responsiveness to different remineralization techniques. Our modified demineralization protocol tailored for primary teeth underscores the importance of methodological adaptation to specific dental substrates [ 20 ]. Additionally, the inclusion of Xylitol in Group II, known for facilitating Ca2+ movement and accessibility, likely contributed to the observed deeper remineralization.

The Nano-P, containing bioactive nano-hydroxyapatite with a high fluoride ion concentration, proved effective in creating a supersaturated environment that promotes mineral re-precipitation within demineralized enamel lesions, penetrating deeper into subsurface layers compared to conventional fluoride treatments [ 37 - 38 ].

The homogeneous layer of precipitated hydroxyapatite on the matrix acts as a scaffold for the deep deposition of nHAP within the prismatic nano-gaps. When compared to fluoride, calcium phosphate nano-crystals of nHAP may penetrate deeper into the subsurface layer of carious lesions, making it more effective than 22,600-ppm fluoride ion in NaF varnish alone, which tends to remain on the surface of the initial layer [ 44 - 45 ].

Nano-P was superior to fluoride alone in our study. However, according to a recently published systematic review, while promising results have been highlighted for the combination of nHAP and fluoride in the remineralization treatment of white spot lesions, the review also notes that the limited number of studies and types of products available prevent recommending widespread use of this combination at this time. The review indicates that the evidence found is of moderate quality, necessitating further research before definitive conclusions can be drawn [ 46 ].

In conclusion, our study underscores the efficacy of combined Nano-P and laser therapies in promoting remineralization of primary tooth enamel. Future research should delve deeper into the mechanisms underlying these synergistic effects to optimize dental treatments tailored to the unique characteristics of primary teeth. Such insights could significantly advance pediatric dentistry by offering more effective strategies for preventing and treating early childhood caries.

While acknowledging the limitations of our *in vitro* design and short-term duration, future studies should investigate the antibacterial properties and long-term safety implications of Nano-P, plasma, and laser therapies to better inform their clinical application. 

## Conclusion

Using a controlled, bacteria-free pH cycling model, this study investigated the synergistic effects of a commercial Nano-P containing 9000 ppm fluoride in conjunction with a low-power 940 nm diode laser, as well as CAP, or Nano-P alone. The results demonstrated significant potential for enhancing remineralization processes, suggesting these treatments can facilitate remineralization even with reduced fluoride concentrations. However, further studies are needed to evaluate their efficacy specifically in primary dentition.
